# The Possibility of Traditional Chinese Medicine as Maintenance Therapy for Advanced Nonsmall Cell Lung Cancer

**DOI:** 10.1155/2014/278917

**Published:** 2014-08-04

**Authors:** Weiru Xu, Guowang Yang, Yongmei Xu, Qing Zhang, Qi Fu, Jie Yu, Mingwei Yu, Wenshuo Zhao, Zhong Yang, Fengshan Hu, Dong Han, Xiaomin Wang

**Affiliations:** Oncology Department, Beijing Hospital of Traditional Chinese Medicine Affiliated to Capital Medical University, 23 Meishuguanhou Street, Dongcheng District, Beijing 100010, China

## Abstract

Lung cancer has become the leading cause of cancer deaths, with nonsmall cell lung cancer (NSCLC) accounting for around 80% of lung cancer cases. Chemotherapy is the main conventional therapy for advanced NSCLC. However, the disease control achieved with classical chemotherapy in advanced NSCLC is usually restricted to only a few months. Thus, sustaining the therapeutic effect of first-line chemotherapy is an important problem that requires study. Maintenance therapy is given for patients with advanced NSCLC if three is no tumor progression after four to six cycles of first-line platinum-based chemotherapy. However, selection of appropriate maintenance therapy depends on several factors, while traditional Chinese medicine (TCM) as maintenance therapy is recommended for all kinds of patients. It has been demonstrated that TCM can prolong the survival time, improve the quality of life (QOL), and reduce the side effects for advanced NSCLC. Although the trials we searched about TCM serving as maintenance therapy is only 9 studies, the results indicate TCM can prolong the progression free survival (PFS) and improve the QOL. So it is possible for TCM to be as maintenance therapy for advanced NSCLC. More rigorous trials are required to further verify its efficacy.

## 1. Introduction

Lung cancer has become the leading cause of cancer deaths in both men and women [1, 2]. Nonsmall cell lung cancer (NSCLC) accounts for around 80% of lung cancer cases [3]. At diagnosis, approximately 70% of patients present advanced stage of malignancy, for which curative therapy will not be available. Chemotherapy, radiotherapy, and targeted therapy are the conventional treatment for advanced NSCLC, among which chemotherapy is the main one. Platinum based doublets chemotherapy is the standard of care for advanced NSCLC. However, the disease control achieved with classical doublets chemotherapy in advanced NSCLC is usually restricted to only a few months [4–6]. About 20–80% of NSCLC patients cannot receive second-line chemotherapy for multiple reasons, including poor compliance [7]. Maintenance therapy can suppress disease progression and provide the opportunity to receive additional treatment. Thus, sustaining the therapeutic effect of first-line chemotherapy is an important problem that requires study. In recent years, maintenance therapy has become a new treatment strategy that aims to sustain a reduced tumor size and relieve tumor-related symptoms, in contrast to conventional chemotherapy that aims to maximize tumor cell death [7].

Maintenance therapy is an option in the National Comprehensive Cancer Network (NCCN) Guidelines only for responding and stable disease patients. Many clinical studies of multiple regimens and modalities about maintenance therapy are currently underway, which has been shown to improve the progression free survival (PFS) [8, 9]. However, some concerns remain regarding the overall survival (OS) and quality of life (QOL) [10]. The application of these chemotherapeutic drugs and molecular targeted drugs in maintenance therapy increase the financial burden of cancer treatment, which is another concern of this therapy.

Traditional Chinese medicine (TCM) has increasingly become popular in the west including in cancer patients [11]. It is estimated the United States National Cancer Institute (NCI) spends around $120 million each year on complementary and alternative medicine (including TCM) related research projects [12]. It has been demonstrated that TCM can alleviate the clinical symptoms, improve the QOL, and reduce the side effects [13]. It helps NSCLC patients to “*survive with tumor*.” So TCM is very suitable for maintenance therapy. In fact, TCM is widely used for NSCLC patients as consolidation therapy which actually includes TCM maintenance therapy. The difference is that maintenance therapy is between first-line and second-line therapy. TCM is endowed with new meaning as the introduction of the concept of maintenance therapy. If the disease is not progressed after first-line therapy, the tumor will be suppressed temporarily. The progression of the tumor is inevitable as time goes by. It is possible to stabilize the tumor, prolong the time to progression, and improve the QOL given with TCM maintenance therapy. Moreover, maintenance therapy is only recommended for patients with performance status (PS) 1-2, while TCM maintenance therapy is recommended for all kinds of patients no matter PS 1-2 or PS 3-4. So it is possible for TCM to be as maintenance therapy for advanced NSCLC.

## 2. The Present Situation of Maintenance Therapy

Maintenance therapy refers to systemic therapy that is given for patients with advanced NSCLC if the response is complete response (CR), partial response (PR), or stable disease (SD) after four to six cycles of first-line platinum-based chemotherapy [14]. The theoretical foundation of this therapy originates from the Goldie-Coldman theory [15], resistant and slowly growing cancer cells remain after first-line chemotherapy which has killed the sensitive and rapidly proliferating cells. Use of different non-cross-resistant chemotherapy regimens is effective in eradicating the remaining resistant cancer cells. Maintenance therapy can be classified into two types: continuation maintenance therapy and switch maintenance therapy. Continuation maintenance therapy refers to the use of at least one of the agents that was given in the first-line regimen. Switch maintenance therapy refers to the initiation of a different agent that was not included as part of the first-line regimen. Selection of appropriate maintenance therapy depends on several factors such as histologic type, performance status, and genetic alternations. NCCN version 2.2013 recommends continuation maintenance therapy using bevacizumab (category 1), cetuximab (category 1), pemetrexed (category 1), bevacizumab plus pemetrexed, and gemcitabine and switch maintenance therapy using pemetrexed, erlotinib, and docetaxel (category 2B).

Maintenance therapy is applied for the continuous treatment while patients can tolerate its side effects with favourable QOL. However, continuing cytotoxic agents will result in cumulative toxicity. Maintenance therapy with several cycles of cytotoxic drugs may damage the immune function, lower the QOL, and increase the risk of drug resistance for continuous therapy. Since researchers are more concerned with the acute toxicity accumulation, most clinical trials except large-scale studies did not evaluate QOL [7].

Based on the recent trials and Food and Drug Administration (FDA) approval, the NCCN Panel recommends single-agent pemetrexed as continuation maintenance therapy in patients with nonsquamous NSCLC who are EGFR mutation negative or ALK fusion negative. A recent phase III randomized trial found that continuation maintenance therapy with pemetrexed slightly increased PFS when compared with placebo [16]. Preliminary results suggest that continuation maintenance therapy with pemetrexed also improves overall survival (OS), but drug-related grades 3 to 4 anemia, fatigue, and neutropenia were significantly higher in pemetrexed-treated patients leading to the decrease of QOL [17]. Consequently, more clinical trials are expected to observe the OS and QOL.

The tolerable molecular targeted drugs are very expensive. Today, in the conditions of Chinese medical security system and the high incidence rate of lung cancer, maintenance therapy with targeted drugs is impossible to implement for most Chinese patients with no apparent disease progression after first-line chemotherapy. In addition, targeted therapy is only for those patients with specific genetic alternation [18], which in part limits its clinical application. Thus, maintenance therapy has some limitations to be applied in clinic due to population selection and high expenditure.

Maintenance therapy with chemotherapy or targeted therapy prolongs the PFS of NSCLC patients, but chemotherapy will accumulate the toxicity and increase the risk of drug resistance. The price of targeted therapy is very expensive and only NSCLC patients with specific gene phenotype will benefit. Some clinicians still doubt the efficacy of maintenance therapy, because most studies have not been shown to evaluate the QOL [7]. Owning to its treatment according to different syndromes, TCM is not limited to treat patients with histologic type, PS. or genetic alternations. These shortages of maintenance with chemotherapy or molecular targeted therapy make opportunities for TCM to treat as maintenance therapy.

## 3. The Theoretical Foundation of TCM in the Treatment of NSCLC

Lung cancer belongs to the disease of Feiji, Feiyong, and Xiji in ancient literature of TCM. The main pathogenesis of lung cancer is the deficiency of vital qi and the invasion of toxic pathogen, secondly for phlegm and blood stasis. Climatic pathogens or cancer toxin will invade into the human lung in case of the deficient vital qi and imbalance of yin and yang. The disharmony of lung function results in the obstruction of lung qi, impairment of dispersing and descending, stasis of blood flow, disturbance of body fluids in distribution. The accumulation of body fluids contributes to phlegm coagulation, causing qi stagnation and blood stasis in collaterals. Then the combination of the stagnation of qi, blood, phlegm and toxin gradually results in a mass in lung. Lung cancer is a kind of disease deficient in the whole body, and excessive in the local body. Deficiency in lung is common with qi deficiency, yin deficiency, and deficiency of qi and blood, while excess in lung is common with phlegm coagulation, qi stagnation, blood stasis, and toxin retention.

TCM holds that the oncogenesis, development and outcome of cancer are the process of the struggle, growth, and decline between vital qi and pathogenic qi in the human body. There are sayings in ancient Chinese medical literature* Yellow Emperor's Inner Classic *“*the region where pathogenic factors invade must be deficient in qi*,” “*when there is sufficient vital qi inside, pathogenic factors have no way to invade the body*.” The oncogenesis is based on the theory of “*internal deficiency.*” As mentioned by Professor Rencun Yu the oncogenesis and extrinsic factor are the condition, the decisive factor is the intrinsic factor of* Internal Deficiency* [19]. The vital qi can sustain the normal physiological functions and defense the pathogenic factors. If the vital qi is deficient, the pathogenic qi will invade. When the stability of human internal environment and the balance between internal and external part are destroyed, the cancerigenic factor will take effect and contribute to oncogenesis, invasion, progression, and metastasis. So the oncogenesis was described in* Yi Zong Bi Du* (*Essential Readings for Medical Professionals*) as “*deficient vital qi leading to the lingering of pathogenic qi is the cause of cancer.*” Furthermore, the consumption caused by cancer itself and continuous treatment (operation, chemotherapy, and radiotherapy) will further injure the qi and blood. The cytotoxic chemotherapy fights against the pathogen post first-line chemotherapy for advanced NSCLC leading to the balance of vital qi and pathogenic qi in a short period of time. Whether the tumor can be controlled or not will be decided by the balance of the human internal environment. When the balance is broken, the tumor will relapse or progress. Thus, the maintenance therapy after first-line chemotherapy for advanced NSCLC obeys the rule of strengthening body resistance and eliminating pathogenic factors. Strengthening body resistance to restore its normal function in main combined with TCM of detoxication and anticancer is the principle to balance between the tumor and the internal environment. When the vital qi restores, it may inhibit the cancer cells, improve the QOL, and prolong the survival time.

In recent years, the theory of tumor microenvironment gains more and more attention of scholars which investigates the effects of tumor microenvironment on cancer relapse and metastasis. The Paget's “*Seed and Soil*” theory proposed in 1889 emphasizes the importance of the interaction between the tumor cell and its environment in order for metastasis and relapse to occur. The hypothesis states that cancer cells can seed to other tissue and change its surrounding cell quality by blood vessels and lymph. The metastasis site is not randomized, but some specific organs with the environment are suitable for the growth of cancer cells. The study shows that the primary tumor has been preparing the “soil” before metastasis so as to build its living environment. There is dynamic balance between the normal cells and its surrounding environment which regulates the cell activity, generation, differentiation, apoptosis, secretion, and expression of cell surface factors. While the oncogenesis of tumor is to break this balance, the tumor will establish the external environment to proliferate infinitely. This process runs through the whole procedure of tumor progression that is the root of oncogenesis and metastasis. This tumor microenvironment is similar to the TCM theory* balance of yin and yang* in human body. The theory of TCM holds that the pathogenesis of all diseases is caused by the imbalance of yin and yang in the body. It is recorded in* Su Wen *(*Plain Questions of the Yellow Emperor's Inner Classic*) that “*If yin and yang are in a relative equilibrium, life activities will be normally maintained; if yin and yang are separated, exhaustion of vital essence will happen*.” The balance of yin and yang correlates with the harmony of the joint functions of each system, the Zang organs in particular. If the human body is balanced, people remain healthy. Otherwise, diseases or even death will occur. Just as the origins of all the diseases, the cause of cancer is also induced by the imbalance of yin and yang, which is manifested as the qi stagnation, phlegm coagulation, blood stasis, and retention of toxic heat. If the imbalance of yin and yang continues, “*yin may separate from yang*,” and vital essence will be exhausted, leading to an ultimate stage of cancer. The imbalance of yin and yang is throughout the whole cause of cancer. The process of oncogenesis, progression, relapse and metastasis is the uninterrupt break of the whole or local yin and yang. So treatment principle is to restore the balance of yin-yang. As was proposed by two famous Chinese oncologists Rencun Yu and Jiaxiang Liu, the cancer treatment should follow the principle of regulating yin and yang to stabilize the internal environment and reestablish the internal balance. Then tumor can be controlled, the vital qi can be protected, the symptoms relieved, the QOL improved, and the survival time prolonged [20, 21]. The* wholism theory*, syndrome differentiation, and treatment in TCM correlate with the theory of tumor microenvironment from the macroscopic or microscopic angles. Recently, more and more scholars start to study the relationship between the TCM theory and tumor microenvironment. The internal environment is in the condition of the balance of yin and yang after first-line chemotherapy with tumor response or stable disease. This condition will be disturbed at any time nevertheless. TCM has an advantage of regulating yin and yang, balancing vital qi and pathogenic qi. Moreover, the therapeutic effects of TCM in the treatment of cancer are definite. So TCM as maintenance therapy for advanced NSCLC is to sustain this balance as long as possible.

## 4. Clinical Trials on TCM Treatment for Advanced NSCLC

TCM shows an advantage in the treatment of advanced NSCLC. It can combine with chemotherapy and radiotherapy that alleviates the side effects, enhances short-term therapeutic effects, stabilizes the disease, reduces the incidence rate of relapse and metastasis, and improves the long-term efficacy [22–25]. As to those patients who cannot accept operation, chemotherapy, or radiotherapy, TCM can ameliorate symptoms, reduce the pain, improve the QOL, and prolong the survival time with tumor [26]. So TCM shows the irreplaceable role in the comprehensive treatment of advanced NSCLC.

In the early 1990s, studies about TCM in the treatment of NSCLC are not much. Chen et al. [27] studied a meta-analysis about TCM in the treatment of NSCLC compared with chemotherapy. It included 14 relevant papers with accumulated 1909 cases from 1990 to 1997. TCM for treating NSCLC in these papers included Chinese patent medicine (see [28–30], etc.), and decoction. The results showed that OR (odds ratios) of TCM group for stable rate (CR + PR + SD) was 2.1 (95% confidence intervals: 0.7–3.08), and OR of chemotherapy group for response rate (CR + PR) was 1.48 (95% confidence intervals: 1.03–2.24). The median survival time (MST) of 7 papers was included. The MST of TCM group was 335.4 days, while that of chemotherapy group was 231.8 days. This study indicates that TCM can prolong the MST, and chemotherapy group shows higher response rate but short MST. However, these studies did not limit the stage of NSCLC which resulted in clinical heterogeneity. More well-designed clinical trials are needed.

Yang et al. [31] reported the therapeutic effects of TCM in the treatment of advanced NSCLC in 2005. 136 cases were randomized into TCM group, integrated group (TCM + chemotherapy), and chemotherapy group. The response rate, syndrome differentiation, QOL, and MST were observed. The patients in both TCM group and integrated group were applied with decoction in combination with* Lanxiangxi Injection *(injection extracted from* Wenchow Turmeric Root Tuber*) and* Elemene Injection* (injection extracted from* Ma-yuen Jobstears Seed*). The results showed that the response rate (CR + PR) for TCM group was 2.1%, for chemotherapy group was 27.2% and for integrated group was 44.4%. The stable rate (CR + PR + SD) in integrated group was higher than other two groups (*P* < 0.05). The clinical symptoms and QOL in integrated group were better than chemotherapy group (*P* < 0.05, *P* < 0.01). The integrated group showed less side effects and toxicity compared to chemotherapy group. The MST in TCM group and integrated group were 11.5 months and 12.3 months, respectively, longer than that in chemotherapy group (9.2 months) (*P* < 0.05). The 1-year survival rates in TCM group and integrated group were 36.1% and 48.9%, respectively, higher than that (27.3%) in chemotherapy group (*P* < 0.05). This study supports that TCM can stabilize the tumor, improve the QOL, prolong the MST and attenuate the toxicity induced by chemotherapy.

A prospective, multicenter, randomized controlled trial was carried out in 6 hospitals [32]. 294 cases of III-IV NSCLC were allocated to TCM group, integrated group (TCM + chemotherapy), and chemotherapy group.* Shenyi Capsule* and* Hechan Pian* combined with decoction were applied to the patients in TCM group and integrated group. The MST of TCM group and integrated group were 292 days and 355 days. respectively, longer than that of chemotherapy group (236 days). The median time to progression (TTP) of TCM group and integrated group were 187 days and 239 days, respectively, longer than that of chemotherapy group (180 days). There was statistical indifference between groups because the sample was not large. But it shows the trend that TCM prolongs MST in III-IV NSCLC.

Zhu and Wu [33] studied a meta-analysis to evaluate the efficacy and safety of specialized TCM prescription integrated with chemotherapy in the treatment of III-IV NSCLC in 2013. Total of 17 randomized controlled trials (RCT) including 1163 cases were included in this meta-analysis. As compared with chemotherapy group, the specialized TCM prescription integrated with chemotherapy group achieved significant benefits in improving clinical response rate and QOL and reducing adverse effects. The ORs for clinical response rate, the improvement of QOL and the rates of adverse effects were 1.44 (95% confidence intervals: 1.12–1.85), 3.36 (95% confidence intervals: 2.45–4.59), and 0.23 (95% confidence intervals: 0.18–0.28), respectively. The results of this meta-analysis implies that the specialized TCM prescription integrated with chemotherapy in the treatment of stages III-IV NSCLC is more effective than chemotherapy alone. But the sample of these trials included is not large. The large sample, multicenter, randomized controlled trials are expected to further verify the efficacy of TCM for advanced NSCLC.

The achievements of TCM in the treatment of cancer have aroused the attention of Chinese government. Supported by our government, many hospitals have begun to develop clinical trials about TCM in the prevention and treatment of cancer. A multicenter, large-sample, randomized, double-blinded controlled trial (National Tenth Five-year Science Project) was led by Guang'an men Hospital, China Academy of Chinese Medical Sciences [22]. 587 nonoperable IIIA-IV NSCLC patients were enrolled. Integrated group (chemotherapy +* Shenyi Capsule* with the effects of tonifying qi and blood) was compared with western medicine group (chemotherapy + placebo). The observation time was two years. Statistical differences were shown in the clinical symptoms, Karnofsky performance status (KPS), chemotherapy induced side effects between integrated group and western medicine group (better in integrated group). The MST of integrated group was 12.03 months which was longer than that of western medicine group (8.46 months). There was statistical difference of MST between integrated group and western medicine group (*P* = 0.0118). This trial implies that integrated therapy can obviously improve the clinical symptoms, alleviate chemotherapy induced side effects and prolong the MST. This study also observes the effects of TCM in the treatment of non-operable IIIA-IV NSCLC patients.* Huachansu Injection*,* Elemene Injection*,* Lanxiangxi Injection* were either alternately applied or alone in TCM group. The MST of TCM group was 10.92 months. The QOL was improved. However, randomization approach was not applied. The latest cohort study supported by National Eleventh Five-year Science Project investigated comprehensive TCM regimen in the treatment of NSCLC [34]. The preliminary results showed that the MST of advanced NSCLC in integrated group was 16.6 months, and that in western medicine group was 13.13 months. These two trials provide high-level, evidence-based medical data for TCM in the treatment of advanced NSCLC. Therefore, it has been demonstrated that TCM alone or combined with conventional therapy in the treatment of advanced NSCLC can prolong the MST, improve the QOL, and reduce side effects.

International society shows a great interest while TCM achieved a series of achievements in the treatment of NSCLC. National Institute of Health collaborates with Guang'an men Hospital, China Academy of Chinese Medical Sciences to study the project Comprehensive TCM Regimen in the Treatment of Advanced NSCLC [26]. Longhua Hospital affiliated to Shanghai University of TCM collaborates with Memorial Sloan-Ketttering Cancer Center for the trial Jinfukang in the Treatment of NSCLC [35]. These international collaborations will provide more convinced evidence for TCM in the treatment of NSCLC.

## 5. Clinical Trials on TCM as Maintenance Therapy for Advanced NSCLC

TCM has a long history in China and is widely applied for cancer treatment. Actually, in clinic advanced NSCLC patients are given with decoction, Chinese patent medicine, injection of Chinese herbal extract, and so forth. If the PS of advanced NSCLC patient is 1-2, conventional therapy combined with TCM will be applied to the patient. TCM will be continuously given to the patients to consolidate the efficacy. If the PS of the patient is 3-4, TCM will be applied to the patient alone. TCM helps patients to relieve symptoms, improve the QOL, reduce the side effects induced by chemotherapy, radiotherapy, or targeted therapy, and prolong the TTP. TCM therapy runs through the whole process of the conventional therapy and is continuously applied after conventional therapy. This kind of consolidation therapy actually includes maintenance therapy. The difference is that maintenance therapy is the intensive treatment between first-line and second-line therapy. So TCM has already acted as the maintenance therapy in China. It has been demonstrated that TCM can in part help patients survive with tumor. So it is very suitable for TCM to be applied as maintenance therapy for advanced NSCLC. As mentioned before maintenance therapy with chemotherapy is only recommended for patients with PS 1-2, while TCM maintenance therapy is recommended for all kinds of patients no matter PS 1-2 or PS 3-4. So it is possible for TCM in the maintenance therapy of advanced NSCLC.

We searched the following sources up to March 2014 using PubMed, CNKI (China National Knowledge Infrastructure), Wanfang Database. Keywords searched were “maintenance therapy” “wei chi zhi liao”, “nonsmall cell lung cancer” “fei xiao xi bao fei ai” or “NSCLC.” No language restriction was applied. After screening titles and/or abstracts, 9 articles were included involving TCM as maintenance therapy in the treatment of advanced NSCLC from the electronic and manual searches. These trials were all conducted in China and published in Chinese. The characteristics of the 9 trials were summarized in Table 1. Most of these trials are small sample, randomized controlled studies. The results indicate that TCM as maintenance therapy can improve the QOL. Part of the studies show that TCM can prolong the PFS compared with the control group (follow-up group). Among them, one study shows that TTP in TCM group is equivalent to that of chemotherapy group, but shows better QOL. Furthermore, there is another study with 162 patients involved from 1992–2007 [36]. It is a nonrandomized controlled study aiming to evaluate the efficacy of TCM as consolidation treatment after conventional therapy. 162 IIIA-IV NSCLC patients after conventional therapy were assigned into TCM group (decoction was applied after conventional therapy) and control group (follow-up after conventional therapy). The results showed that the 2-year and 3-year survival rate of TCM group were much higher than those of the control group (*P* < 0.05). The MST of TCM group was 18 months, while that of control group was 12 months. So it supports that TCM as consolidation therapy can prolong the MST and improve the 2-year, 3-year survival rate. Although the IIIA-IV NSCLC patients after conventional therapy were included into this trial, which did not meet the criteria of maintenance therapy (advanced NSCLC after first-line chemotherapy with no tumor progression), it suggested that advanced NSCLC patients can benefit from TCM consolidation therapy to some degree.

Although the prescriptions (TCM applied) in these trials we mentioned above are different, it demonstrates the feasibility of TCM as maintenance therapy and suggests the profound knowledge of syndrome differentiation. Since previous data shows that TCM serving as maintenance therapy can improve the QOL and PFS. We hope to provide more opportunity for advanced NSCLC patients treated with TCM maintenance therapy to accept second-line therapy after the progression of first-line therapy. More clinical trials are required to further verify its efficacy.

## 6. Prospect on Maintenance Therapy with TCM for Advanced NSCLC

TCM has a long history in the treatment of advanced cancer patients and applied extensively in China. In clinic, TCM is commonly used for advanced NSCLC patients after chemotherapy, which has achieved certain therapeutic effects. This is a kind of maintenance therapy actually. Previous clinical trials have demonstrated that TCM has its own advantage in treating advanced NSCLC compared to western medicine. As we know, the curative therapy for advanced NSCLC is not available. TCM emphasize the* Wholism* and* Survival with Tumor*. TCM regulates the balance of the human body by adjusting the yin and yang, qi and blood, deficiency and excess, which dispels exogenous pathogen by avoiding hurting vital qi. TCM regards the human body more important than the tumor. Although the efficacy of TCM directly in killing and suppressing the tumor cells is not very obvious, it has its own characteristics in some aspects. It can improve the QOL and alleviate the side effects of chemotherapy and radiotherapy. Besides, both the clinical and experimental studies show that TCM especially the tonifying herbal medicine can improve the immune function by elevating the quantity of T-lymphocyte subtypes and natural killer cells, which improves the ability of the human body to fight against tumor cells [37–41]. In 2006, World Health Organization defines cancer as a kind of treatable, controllable chronic disease rather than the incurable disease [42]. This is similar to the idea of TCM* Survival with Tumor.*

Syndrome is the basic unit and a key concept in TCM theory. It is different from disease or symptom. TCM syndrome is the abstraction of a major disharmonious pathogenesis.* Syndrome Differentiation and Treatment* is the core of TCM, which requires treating patients with methods of inspection, listening and smelling, questioning, and pulse taking according to different person, area, and time. Based on this principle, TCM treats with different plans for different stages of cancer patients. Tonifying therapy is mainly for alleviating the side effects induced by chemotherapy and radiotherapy, improving the symptoms and promoting the body recovery. While after the chemotherapy and radiotherapy, dispelling the exogenous pathogen is mainly used for controlling the cancer so as to prevent cancer relapse and metastasis.* Syndrome Differentiation and Treatment* will show its advantage in maintenance therapy. No matter the PS 1-2 or PS 3-4, the gene phenotype, pathology, TCM can serve as maintenance therapy according to different syndromes. During maintenance therapy keeping a favourable living state is the premis, while TCM can improve the QOL and regulates the patient body in a balanced state. So the aim of TCM is similar to maintenance therapy. We believe that TCM as maintenance therapy is feasible.

## 7. Summary

Maintenance therapy with chemotherapy or targeted agents can prolong the PFS of advanced NSCLC patients to some degree, but chemotherapy may increase the toxicity and the risk of drug resistance, and targeted therapy is very expensive and only suited for certain patients with specific genetic alternation [43]. So some patients lose the opportunities to accept maintenance therapy. TCM is widely used in China for cancer patients. Although the effects of TCM in eradicating cancer cells are not obvious, it helps cancer patients to fight against cancer and restore the body into a balanced state by regulating the balance of yin and yang. Besides, TCM can be applied for NSCLC patients not limited in population selection. Recent studies demonstrate that TCM as maintenance therapy can improve the QOL of advanced NSCLC patients. There is some encouraging evidence of TCM for prolonging the PFS. However, there are only small sample clinical trials about TCM as maintenance therapy for advanced NSCLC. More large-scale trials of TCM as maintenance therapy for advanced NSCLC are expected.

## Figures and Tables

**Table 1:** The characteristics of the trials for TCM as maintenance therapy in the treatment of advanced NSCLC.

Authors	TNM stage of NSCLC	Design of study	Patients (*n*)		Intervention		Measurements	PFS, OS, MST, and TTP (m)	Results/conclusion
			Treatment group	Control group	Treatment group	Control group			
Liu et al., 2009 [44]	Nonoperatable IIIA-IV	RCT	31	31	*Feitai Capsule *	—	FACT-L, KPS, clinical symptoms	—	*Feitai Capsule* can improve the QOL.
Chai et al., 2011 [45]	IIIB-IV	RCT	32	32	*Xiaoji Yin *	—	MST, PFS	PFS: 5.1 versus 2.5 (*P* = 0.00) MST: 6.43 versus 3.26 (*P* = 0.00)	*Xiaoji Yin* can prolong the MST and PFS.
Jiang et al., 2011 [46]	IIIB-IV	RCT	25	25	Comprehensive TCM therapy	Gemcitabine/P emetrexed/Do cetaxel	TTP, QOL(EORTC QLQ-LC43)	TTP: 2.9 versus 2.13 (*P* = 0.063)	The TCM comprehensive regimen had equivalent efficacy on TTP but better QOL when compared with single-agent chemotherapy.
Xi et al., 2011 [47]	III-IV	RCT	44	34	*Hechan Pian *	—	PFS, clinical symptoms	median PFS: 5.67 versus 4.12 (*P* = 0.048)	*Hechan Pian* can prolong the median PFS and improve the clinical symptoms.
Zeng et al., 2013 [48]	IV	RCT	34	35	*Shenyi Capsule *	—	Medin PFS, KPS, immune function (T-lymphocyte subtypes)	median PFS: 6.5 versus 6.2 (*P* > 0.05)	*Shenyi Capsule* can improve the QOL and immune function which helps more patients to accept second-line chemotherapy.
Yang 2013 [49]	III-IV	RCT	40	40	*Fuzheng Xiaoji Yin *	—	PFS	PFS: 7.5 versus 4.8 (*P* < 0.01)	*Fuzheng Xiaoji Yin* can prolong the PFS.
Liang and Zhang 2013 [50]	IIIB-IV	RCT	38	36	*Rongyan Capsule *	—	MST, PFS	PFS: 4.8 versus 2.37 (*P* = 0.00) MST: 6.23 versus 2.7 (*P* = 0.00)	*Rongyan Capsule* can prolong the MST and PFS.
Wang et al., 2013 [51]	IV	RCT	39	37	TCM therapy based on syndrome differentiation	—	PFS, QOL(EORTC-QLQ-C30), clinical symptoms	PFS: 7.2 versus 4.4 (*P* < 0.01)	TCM therapy based on syndrome differentiation can improve the QOL, clinical symptoms and prolong the PFS.
Wu et al., 2014 [52]	IIIB-IV	Nonrandomized controlled trial	20	20	*Shenyi Capsule *	—	DCR, PFS, OS	PFS: 4.35 versus 2.67 (*P* < 0.01) OS: 10 versus 7.8 (*P* < 0.01)	*Shenyi Capsule* can prolong the PFS and OS and improve the DCR.

RCT: randomized controlled trial; FACT-L: functional assessment of cancer therapy-lung; MST: median survival time; PFS: progression free survival; TTP: time to progression; QOL: quality of life; EORTC: European Organization for Research and Treatment of Cancer; KPS: Karnofsky performance status; TCM: traditional Chinese medicine; DCR: disease control rate; OS: overall survival.
